# Elevated Pretherapy Serum IL17 in Primary Hepatocellular Carcinoma Patients Correlate to Increased Risk of Early Recurrence after Curative Hepatectomy

**DOI:** 10.1371/journal.pone.0050035

**Published:** 2012-12-05

**Authors:** Jianxiong Wu, Jun Du, Liguo Liu, Qian Li, Weiqi Rong, Liming Wang, Ying Wang, Mengya Zang, Zhiyuan Wu, Yawei Zhang, Chunfeng Qu

**Affiliations:** 1 Department of Surgery, Cancer Institute/Hospital, Chinese Academy of Medical Sciences, Beijing, China; 2 State Key Laboratory of Molecular Oncology, Cancer Institute/Hospital, Chinese Academy of Medical Sciences, Beijing, China; 3 National Office for Cancer Prevention and Control, Cancer Institute/Hospital, Chinese Academy of Medical Sciences, Beijing, China; 4 Environmental Health Sciences Division, Yale University School of Public Health, New Haven, Connecticut, United States of America; Beijing Institute of Infectious Diseases, China

## Abstract

**Background and Aims:**

Primary hepatocellular carcinoma (HCC) is usually presented in inflamed fibrotic/cirrhotic liver with extensive lymphocyte infiltration. We examined the associations between the HCC early recurrence and alterations in serum levels of inflammatory cytokines.

**Methods:**

A cohort of 105 HCC patients with chronic hepatitis B virus infection were included. Pre-therapy, we quantified their serum concentrations of Th1-, Th2-, Th17-, Treg-related, and other cytokines that have been reported to be associated with poor prognosis in human cancers. IL17-producing T-cells were generated *in vitro* from HCC patients and co-cultured with HCC cell lines separated by a 0.4 µM transwell.

**Results:**

All the 105 cases of HCC patients had liver cirrhosis. The patients who suffered from HCC early recurrence had higher pre-therapy serum levels of IL17 and lower levels of IL10 than those who did not suffer from recurrence after curative hepatectomy. After adjustment for general tumor clinicopathological factors, elevated serum levels of IL17 (≥0.9 pg/ml) was found to be an independent risk factor for HCC early recurrence with a hazard ratio of 2.46 (95%CI 1.34–4.51). Patients with bigger tumors (>5 cm in diameter) and elevated serum levels of IL17 had the highest risk of early recurrence as compared to those with only one of these factors (*P* = 0.009) or without any (*P<*0.001). These factors showed similar effects on the HCC patient overall survival. Intrahepatic infiltrated T-cells in HCC patients were identified as the major IL17-producing cells. Proliferation of HCC cells, QGY-7703, was augmented QGY-7703, was augmented in the presence of IL17-producing T-cells. This effect diminished after neutralizing antibody against human IL17A or TNFα was included.

**Conclusion:**

Both tumors and IL17 from liver infiltrated T-cells contributed to HCC early recurrence and progression after curative resection. Pre-therapy serum IL17 levels may serve as an additional indicator for predicting high-risk patients.

## Introduction

Hepatic resection has been the mainstay of curative treatment for primary hepatocellular carcinoma (HCC) that is confined to the liver with satisfactory liver function preserved [1], [2], [3]. However, the high rate of recurrence remains challenging for HCC therapy. According to a recent report, recurrence can occur in as high as 80% of Asian patients within 5 years after resection [4].

Typically, recurrence rates in HCC follow a 2-peak distribution: the early recurrence usually occurs within 2 years after resection and is most closely related to cancer metastasis spread; the late recurrence mainly results from *de novo* tumors as a consequence of the carcinogenic cirrhotic environment [5]. Some clinicopathological factors have demonstrated as the indicators of poor prognosis in HCC, including vascular invasion by tumor, greater tumor size, increased serum levels of alpha fetal protein (AFP), lower grade of tumor differentiation, and tumor multiplicity [1], [2], [3], [4], [5]. Recent molecular studies found that HCC recurrence after hepatic resection could be reasonably predicted, not only by the gene signatures of the tumor tissues themselves [6], [7], but also by that of adjacent non-tumorous liver tissues, including genes related to immune responses [7], [8], [9]

HCC is usually present in inflamed fibrotic or cirrhotic liver with extensive lymphocyte infiltration. Previously, we demonstrated that persistent chronic HBV infection plays a dominant role in HCC in China [10]. Persistent HBV infection is mainly due to inefficient CD4^+^ T cell priming early in the infection and subsequent development of a quantitatively and qualitatively ineffective CD8^+^ T cell responses [11]. Specific Th1-related immunity to HBV viral antigens was found to be defective in chronic HBV infection. Alternatively, there was found to be a large influx of non-virus-specific T cells into the liver [12]. CD4^+^T helper (Th) cells are the master regulators of adaptive immune responses that control different types of pathogen infections and regulate disease progression. These cells play regulatory roles mainly through secreted cytokines [13]. Studies on some cancers, such as lung cancer, have demonstrated that immune-related cytokine gene signature of the tumor surrounding tissues can predict disease progression, and their serum levels are correlated with overall survival [14], [15].

As reported the HCC patients survival is positive associated with higher expression of a group of inflammatory and innate immune genes within the tumors [16]. Molecular analysis found that HCC recurrence after hepatic resection can be well predicted by the gene signatures of adjacent nontumorous liver tissues, including genes related to immune responses [7], [8], [9]. It is yet unclear whether the alterations in serum levels of some inflammatory cytokines are related to HCC recurrence following curative hepatectomy. In this study, we determined the pre-surgery serum levels of some inflammatory cytokines and examined the associations between the HCC early recurrence and alterations in serum levels. Further, we investigated their effect on HCC cell proliferation *in vitro*.

## Materials and Methods

### Ethics Statement

The study protocol (CH-BMS-002) for collecting and using human samples was approved by the Institutional Ethics Committee of Cancer Institute/Hospital, Chinese Academy of Medical Sciences. A written informed consent was obtained from all participants involved in this work.

### Patients

A cohort of 105 cases had HBV-related HCC accompanied by liver cirrhosis from the Cancer Institute/Hospital, Chinese Academy of Medical Sciences in Beijing, were included in this study. Curative surgical resections were performed between April 2007 and October 2010. The following criteria were met based on the guidelines for HCC management distributed by the Chinese Society of Clinical Oncology: 1) pathologically confirmed primary HCC with no extrahepatic metastasis; 2) well-preserved liver function, Child-Pugh class A; 3) no other therapy such as transarterial chemoembolization or radiofrequency ablation prior to surgery; 4) no thrombus in main portal vein or inferior vena cava; 5) no death postoperatively, defined as all deaths within 30 days or during the same hospital stay post-surgery [17]. Curative resection was performed as reported [18]; this is defined as removal of all recognizable tumor tissue. The absence of tumor cells along the parenchymal transection line (cut surface) was confirmed pathologically. General information on the 105 HBV related HCC patients is given in Table 1.

**Table 1:** General information of 105 cases of HBV related HCC patients.

Category	Subcategory	Value, n (%)
Age (year)	median (IQR)	53 (46–60)
Gender	Male	91 (86.7)
	Female	14 (13.3)
HBV infectious status	HBeAg (-)	77 (73.3)
	HBeAg (+)	28 (26.7)
Serum HBV-DNA level	<10000 copies/mL	57 (54.3)
	≥10000 copies/mL	48 (45.7)
Serum AFP	≤100 ng/mL	67 (63.8)
	>100 ng/mL	38 (36.2)
ALT	<40 U/L	67 (63.8)
	≥40 U/L	38 (36.2)
Total bilirubin	<17.1 uM/L	84 (80.0)
	≥17.1 uM/L	21 (20.0)
Albumin	<40 g/L	37 (35.2)
	≥40 g/L	68 (64.8)
Child-Pugh class	A	105 (100)
	B	0 (0)
BCLC classification*	0	4 (3.8)
	A	83 (79.0)
	B	18 (17.1)
Resection	Minor	80 (76.2)
	Major	25 (23.8)
Surgical Margin	≥2 cm	77 (73.3)
	<2 cm	28 (26.7)
Tumor size in diameter	≤5 cm	64 (61.0)
	>5 cm	41 (39.0)
Tumor numbers	1	87 (82.9)
	≥2	18 (17.1)
Edmondson-Steiner grade	I-II	74 (70.5)
	III-IV	31 (29.5)
Microvascular invasion	No	92 (87.6)
	Yes	13 (12.4)

* Barcelona Clinic Liver Cancer classification.

All patients were followed at regular intervals after surgery, every 3 months for the first 2 years and every 6 months for the next 3 years. In each of the follow-up visits, liver function tests, AFP measurement, ultrasonography on the abdomen, and chest radiography were performed. Computer tomography (CT) or magnetic resonance imaging (MRI) was also performed, if deemed necessary. Tumor recurrence was recorded when a tumor was detected by ultrasonography, CT or MRI. Patients with recurrent tumors were treated according to the guidelines for HCC management distributed by the Chinese Society of Clinical Oncology [19]. Disease-free survival time (DFS) was defined as the period from the date of the hepatectomy to the date of detection of the tumor’s recurrence. Follow-up on all patients was concluded by December 2011. The overall survival (OS) time was defined as the period from the date of therapy till death or the end of follow-up.

### Quantification of Serum Levels of Cytokines

Serum cytokine concentrations may be influenced by both infection and surgical procedures. Therefore, serum samples from all the 105 patients were collected prior to therapy when the patients had no symptoms of infection other than HBV or HCV. We measured serum concentrations of Th1-, Th2-, Th17-, and Treg-related, as well as other cytokines that have been reported to be associated with poor prognosis in other types of tumors [15]. All cytokines analyzed were undetectable in sera from seven healthy volunteers. Th1-related (IL2, IL12, IL15, TNF-β) and Th2-related (IL4, IL5, IL13) cytokines were below the detection levels in 36 HCC and 36 chronic HBV infected patients when we first used the Quansys human 16-pix assay kits. Therefore, for the other samples we did not attempt to determine the levels of these cytokines: IL2, IL12, IL15, TNF-β, IL4, IL5, IL13.

The following cytokines: IL-1α, IL-1β, IL6, IL8, IL10, IL17, IL23, IFN-γ and TNF-α in serum were assayed using the Quansys human 9-plex assay kit (Quansys Biosciences, Utah, USA). This was performed according to the manufacturer’s instructions. Serum samples were 1∶2 diluted and conducted in duplicate. Final concentration of each cytokines was calculated based on standard curves by using the Q-view software (Quansys Biosciences, Utah, USA).

### Intracellular Staining of IL17 and Flow Cytometry Analysis (FACS)

Peripheral blood mononuclear cells (PBMCs) were prepared by density gradient centrifugation on Ficoll (Haoyang Biological Company, Tianjin, China) from 3 ml of heparinized blood using standard laboratory protocol as our previous report [20]. Intrahepatic lymphocytes (IHL) were prepared as reported [21]. Approximately 200 mg of tumor adjacent liver tissues (1.5-2 cm away from tumor edges) were cut into 1–2 mm^3^ and digested in 3 ml of liver digestion medium (LDM, Gibco, in vitrogen, USA) for 30 minutes. The IHL were further prepared by density gradient centrifugation on Ficoll. The PBMCs or IHL were incubated with plate-bound mouse anti-human CD3 (10 µg/ml) and 2 µg/ml of anti-human CD28 for 16 hours in RPMI 1640 medium supplanted with 10% fetal calf serum. Then 0.4 µM of Monensin (BD Pharmingen, CA, USA) was added during the last 4 hours of incubation. As parallel, 50 ng/ml of phorbol 12-myristate 13-acetate (PMA) and 1 µM of ionomycin (Sigma, St. Louis, MO, USA) were used as stimulator in the presence of 0.4 µM Monensin for 4 hours as reported [22] in 3 of the patients. The cells were collected and stained with PE-conjugated anti-mouse IgG, followed by staining with PE-Cy5 conjugated anti-human CD4, and PE-Cy7 conjugated anti-human CD8. After the surface staining, the cells were fixed and permeabilized (BD Pharmingen, CA, USA). FITC-conjugated anti-human IL17 was added. All the antibodies used here were purchased from eBioscience (San Diego, CA, USA). The data were acquired in LSR-II (BD, CA, USA) and analyzed using Flowjo software (Tristar, CA, USA).

### Co-culture of HCC Cells with IL17-producing Cells Isolated from HCC Patients

The HCC cell line Hep-3B was purchased from ATCC, USA; QGY-7703, which was described in reference [23], [24], was purchased from Type Culture Collection of Chinese Academy of Sciences, Shanghai, China. Restricted by ethics, we failed to get enough amounts of fresh sectioned liver tissues for isolating the IL17-producing T cells to co-culture with their own tumor cells or to co-culture with the HCC cell line. IL17-producing T cells were generated from PBMCs of HCC patients as described [25]. Briefly, PBMCs were isolated from 10 ml of peripheral blood and cultured with recombinant human IL-23 (20 ng/ml, R&D systems, MN, USA) in the presence of 10 µg/ml of plate-bound anti-human CD3 and 1 µg/ml of anti-human CD28 (all were purchased from eBioscience, CA, USA) for 7–12 days. Presence of IL17-producing T cells in the cultured PBMCs was confirmed by FACS staining.

Approximately 2×10^4^ Hep-3B or QGY-7703 cells were added to the bottom of a 24-well plate. Around 1×10^5^ cultured PBMCs were added to the upper chambers of a 0.4 µm transwell (Corning, NY, USA). In order to activate the T cells, anti-human CD3 and CD28 (5 µg/ml each) were included in the medium. In some co-culture system, 5 µg/ml anti-human IL-17A or 5 µg/ml anti-human TFNα neutralizing antibodies (all were from eBioscience, CA, USA) were included in the medium. After being co-cultured for 48 h, the upper chambers (containing differentiated PBMCs) were removed. Proliferation of the HCC cells was determined using a Cell Counting Kit-8 (Dojindo, Japan) in accordance with the manufacturer’s instructions. Differences in cell proliferation between treatment groups were compared using paired t-tests.

### Statistical Analysis

Continuous variables were expressed as median and interquartile range (IQR). The univariate or multivariate Cox proportional hazard models were used for evaluating the association between clinicopathological factors and serum cytokines and HCC recurrences. The Mann-Whitney U test was used to compare the differences in serum cytokine concentrations between the groups of HCC recurrence and non-recurrence. Spearman’s correlation was used to analyze the correlation between different types of serum cytokines. The cutoff points for serum cytokines were determined using receiver operating characteristic (ROC) curves and the Youden Index. The DFS curves and cumulative OS curves were estimated by the Kaplan–Meier method and compared using the Log-Rank test. SPSS software, version 15.0 (IBM Corporation) was used for all analyses. All *P* values were two-tailed, and the significance level was specified as *P<*0.05.

## Results

### Pre-surgery Serum Concentrations of Inflammatory Cytokines in Patients with and without HCC Early Recurrence after Curative Hepatectomy

From the date of surgery, the mean and median follow-up times for the cohort of 105 cases of HBV-related HCC were 26 and 20 months, respectively. Among them, 60 cases (57.1%) suffered from early recurrence (within 24 months after surgery), of whom 58 (96.7%) patients were intrahepatic recurrence.

Compared to the group of patients without HCC early recurrence (Table 2), patients with HCC early recurrence had significantly higher serum concentrations of IL17 *(P<0.001)* and lower serum concentrations of IL10 (*P = 0.005*). Serum levels of the other cytokines including IL23, IFN-γ·, IL8, IL-1β, IL6, IL-1α and TNF-α were similar between the two groups of patients with and without HCC early recurrence. Spearman’s correlation test indicated that serum concentrations of IL17 and IL10 were borderline significantly correlated (*R =  - 0.192, P = 0.0496).*

**Table 2:** Pretherapy serum levels of cytokines in patients with and without HCC recurrence.

Cytokines	Early recurrence a	Non-recurrence a	*P* value^a^
IL17 (pg/ml)	1.45 (0.50–2.48)	0.30 (0.20–0.50)	<0.001^b^
IL10 (pg/ml)	4.65 (3.30–6.70)	7.00 (4.10–15.55)	0.005^b^
IL23 (pg/ml)	39.80 (0.00–3612.00)	0.00 (0.00–1061.00)	0.132
IFNγ (pg/ml)	0.20 (0.10–0.40)	0.10 (0.00–0.40)	0.134
IL8 (pg/ml)	31.65 (6.98–90.55)	38.40 (12.30–324.00)	0.169
IL-1β (pg/ml)	4.65 (3.60–5.98)	3.60 (2.95–5.50)	0.202
IL6 (pg/ml)	0.05 (0.00–5.40)	0.50 (0.00–19.50)	0.299
IL-1α (pg/ml)	0.15 (0.00–1.80)	0.00 (0.00–1.60)	0.750
TNFα (pg/ml)	3.00 (1.13–6.48)	2.50 (1.05–9.05)	0.823

a cytokine levels are expressed as median and interquartile range, *P* values are based on Mann-Whitney U test.

b Spearman’s correlation test, *R = * - 0.192, *P* = 0.0496 between the serum levels of IL17 and IL10.

We further analyzed whether the serum concentrations of IL17 and IL10 were associated with tumor clinicopathological factors that were generally accepted as indicators of higher risk for HCC recurrence, including tumor size, tumor multinodularity, presence of microvascular invasion, lower grading of the tumor, and high serum level of AFP. No significant associations were observed between them (Table 3).

**Table 3:** Associations between the pretherapy serum levels of IL17 and IL10 and general clinicopathological factors in HCC patients.

		IL17		IL10	
Category	Subcategory	median (IQR)	*P* value*	median (IQR)	*P* value*
Microvascular invasion	No (n = 92)	0.50 (0.20–1.75)	0.433	4.80 (3.50–9.08)	0.433
	Yes (n = 13)	0.60 (0.45–1.45)		4.80(4.65–25.20)	
Tumor size (cm)	≤5 (n = 64)	0.50 (0.20–1.50)	0.279	4.80 (3.50–9.08)	0.452
	>5 (n = 41)	0.60 (0.30–1.85)		4.80 (3.80–10.45)	
Tumor number	1 (n = 87)	0.50 (0.20–1.60)	0.618	4.80 (3.50–8.70)	0.956
	≥2 (n = 18)	0.55 (0.38–1.65)		5.25 (3.60–10.78)	
Edmondson-Steiner grade	I-II (n = 73)	0.50 (0.20–1.65)	0.609	5.00 (3.45–10.55)	0.631
	III-IV(n = 32)	0.70 (0.30–1.60)		4.75 (3.73–7.15)	
Serum AFP level (ng/mL)	≤100 (n = 67)	0.50 (0.20–1.50)	0.110	5.00 (3.80–9.80)	0.214
	>100 (n = 38)	0.80 (0.30–1.80)		4.70 (3.30–8.30)	

* Mann-Whitney U test.

### HCC Early Recurrence Risk after Curative Hepatectomy in Patients with Increased Serum IL17 and Decreased IL10

We then analyzed the recurrence risk of HCC in relation to serum levels of IL17 and IL10 after adjusting for general clinicopathologic factors. The cutoff values of IL17 and IL10 were based on the ROC curves (Figure S1). The area under curve (AUC) for IL17 was 0.824 *(P<0.001)* with the cutoff value of 0.9 pg/ml; it was 0.662 *(P = 0.003)* for IL10 with a cutoff value of 8.2 pg/ml.

Among the 105 patients, 42 (40. 0%) cases had elevated serum IL17 (IL17≥0.9 pg/ml), and 38/42 of these cases suffered from early recurrence; 73 (69.5%) cases had decreased serum IL10 (IL10<8.2 pg/ml), and 49/73 (67.1%) cases experienced early recurrence (Table 4). Compared to those with serum IL17<0.9 pg/ml, patients with serum IL17≥0.9 pg/ml had significantly increased risk of HCC early recurrence (adjusted HR = 2.46, 95% CI 1.34–4.51). Decreased serum IL10 (<8.2 pg/ml) alone showed marginal effect (crude HR = 1.91, 95% CI 0.99–3.71) on early recurrence, the risk, however, was diminished after adjust for serum IL17 levels and clinicopathologic factors (adjusted HR = 1.40, 95% CI 0.68–2.89) (Table 4).

**Table 4:** Associations between HCC early recurrence and serum levels of IL17 and IL10, as well as some selected clinicopathological factors in HCC patients#.

Category	Subcategory	Recurrence	Non-recurrence	Crude HR^a^	Adjusted HR^b^
Serum IL17	<0.9 pg/ml	22	41	1.00	1.00
	≥0.9 pg/ml	38	4	2.39 (1.39–4.13)*	2.46 (1.34–4.51)*
Serum IL10	≥8.2 pg/ml	11	21	1.00	1.00
	<8.2 pg/ml	49	24	1.91 (0.99–3.71)	1.40 (0.68–2.89)
Tumor size	≤5 cm	32	32	1.00	1.00
	>5 cm	28	13	1.95 (1.16–3.30)*	2.00 (1.09–3.68)*
Tumor grade	I-II	40	34	1.00	1.00
	III-IV	20	11	1.75 (1.01–3.04)*	1.59 (0.86–2.94)
Tumor number	1	48	39	1.00	1.00
	≥2	12	6	1.61 (0.85–3.05)	1.91 (0.90–4.06)
Vascular invasion	No	51	41	1.00	1.00
	Yes	9	4	1.87 (0.91–3.84)	1.37 (0.58–3.27)
Serum AFP	≤100 ng/mL	33	34	1.00	1.00
	>100 ng/mL	27	11	1.44 (0.86–2.41)	1.16 (0.67–2.01)
HBV status	HBeAg (-)	18	10	1.00	1.00
	HBeAg (+)	42	35	1.21(0.69–2.13)	1.22(0.65–2.32)

# The other clinicopathological factors on HCC early recurrence was presented in Table S1.

a HR: hazard ratio.

b Adjusted for age, gender and the other factors included in this table.

* *P<0.05.*

### HCC Early Recurrence Risk after Curative Hepatectomy in Patients with the Presence of General Tumor Clinicopathological Factors

In this cohort, the risk of HCC recurrence was also significantly associated with bigger tumor size (>5 cm in diameter, crude HR = 1.95, 95% CI 1.16–3.30) and tumor poor differentiation (Edmondson-Steiner grade III-IV, crude HR = 1.75, 95% CI 1.01–3.04) (Table 4). However, after adjusting for serum levels of IL17, IL10, as well as the other general clinicopathological factors, the risk remained statistically significant for tumor size only (adjusted HR = 2.00, 95% CI 1.09–3.68) (Table 4).

Vascular invasion, which has been suggested by others to be the strongest predictor of HCC early recurrence [5], showed suggestive effects on recurrence in this cohort (adjusted HR = 1.37, 95% CI 0.58–3.27). More than one tumor numbers (adjusted HR = 1.91 95%CI 0.90–4.06) appeared to have a suggestive effect on early recurrence. Status of serum HBeAg positive (adjust HR = 1.22, 95%CI 0.65–2.32) and higher serum levels of AFP (>100 ng/ml, adjusted HR = 1.16, 95%CI 0.67–2.01) were not significantly associated with the early recurrence (Table 4). The associations with HCC early recurrence were also appeared not significant for the other general clinocopathological factors, including serum HBV-DVA copies, ALT, total bilirubin, albumin levels and Barcelona Clinic Liver Cancer (BCLC) Classification, as well as surgical margin, the way of resection and blood transfusion (presented in Table S1).

### HCC Recurrence Risk with Increased Serum IL17 and Bigger Tumor Size

**Figure 1:**
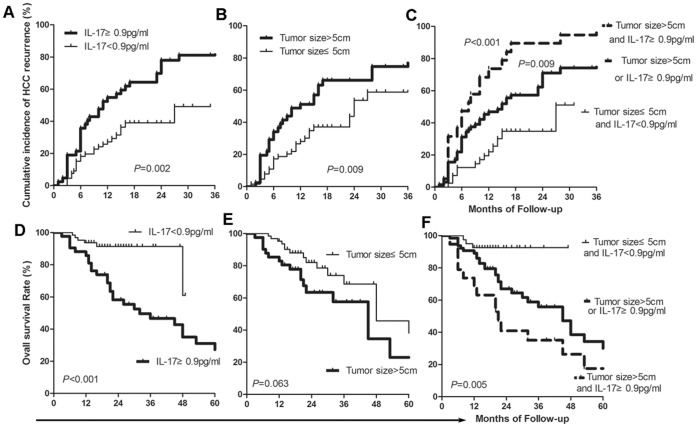
Effects of serum levels of IL17 and tumor size on HCC early recurrence (A,B,C) and overall survival of the patients (D,E,F). Kaplan-Meier estimate on HCC recurrence based on elevated serum levels of IL17 alone (A), on tumor size alone (B), and on combination of the two factors (C). Kaplan-Meier estimate patient overall survival based on elevated serum levels of IL17 alone (D), on tumor size alone (E), and on combination of the two factors (F).

Given the contribution to HCC early recurrence from bigger tumor size and the elevated serum IL17 levels we combined the two factors to examine the risk of early recurrence using the Kaplan-Meier method (Figure 1). Patients with elevated serum IL17 levels (IL17≥0.9 pg/ml) showed significantly higher rates of early recurrence than those with serum IL-17<0.9 pg/ml (*P* = 0.002) (Figure 1A). Patients with tumors larger than 5 cm in diameter showed significantly higher rates of early recurrence than those with smaller tumors (≤5 cm) (*P* = 0.009) (Figure 1B). Serum IL17 level appeared to be more significant when predicting recurrence than relative tumor size. It was clear that patients with both factors simultaneously had the highest rate of early recurrence compared to those with only one factor (*P = *0.009) or those with neither (*P<*0.001) (Figure 1C). The sensitivity and specificity of these two factors in predicting HCC early recurrence is provided in Table 5.

**Table 5:** Sensitivity and specificity of elevated pre-therapy serum IL17 and bigger tumor size in predicting HCC early recurrence and overall survival.

	Early recurrence		overall survival	
	Sensitivity(%)	Specificity(%)	Sensitivity(%)	Specificity(%)
Serum IL17≥0.9 pg/mL	63.3	91.1	79.0	77.4
Tumor size>5cm	46.67	71.1	52.6	65.5
One of the factors	78.3	62.2	86.8	50.0
Both of the factors	31.7	100	44.7	92.9

We further analyzed the effect of bigger tumor size in combination with increased serum levels of IL17 in predicting HCC progression (Figure 1D, 1E, 1F). Kaplan-Meier survival analysis demonstrated that both elevated serum levels of IL17 (*P* = 0.001, ≥0.9 pg/ml verse <0.9 pg/ml) (Figure 1D) and bigger tumor size (*P* = 0.041, >5 cm verse≤5 cm in diameter) (Figure 1E) reasonably predicted the OS of HCC patients. When the two factors were combined, patients with neither of the two factors showed much better progression than those with both factors (*P* = 0.002) or those with either one (*P* = 0.033, Figure 1F). The sensitivity and specificity of these two factors in predicting HCC early recurrence is provided in Table 5.

### Intrahepatic CD4^+^ T and CD8^+^ T Cells were Identified as the Mainly IL17- Producing Cells

**Figure 2:**
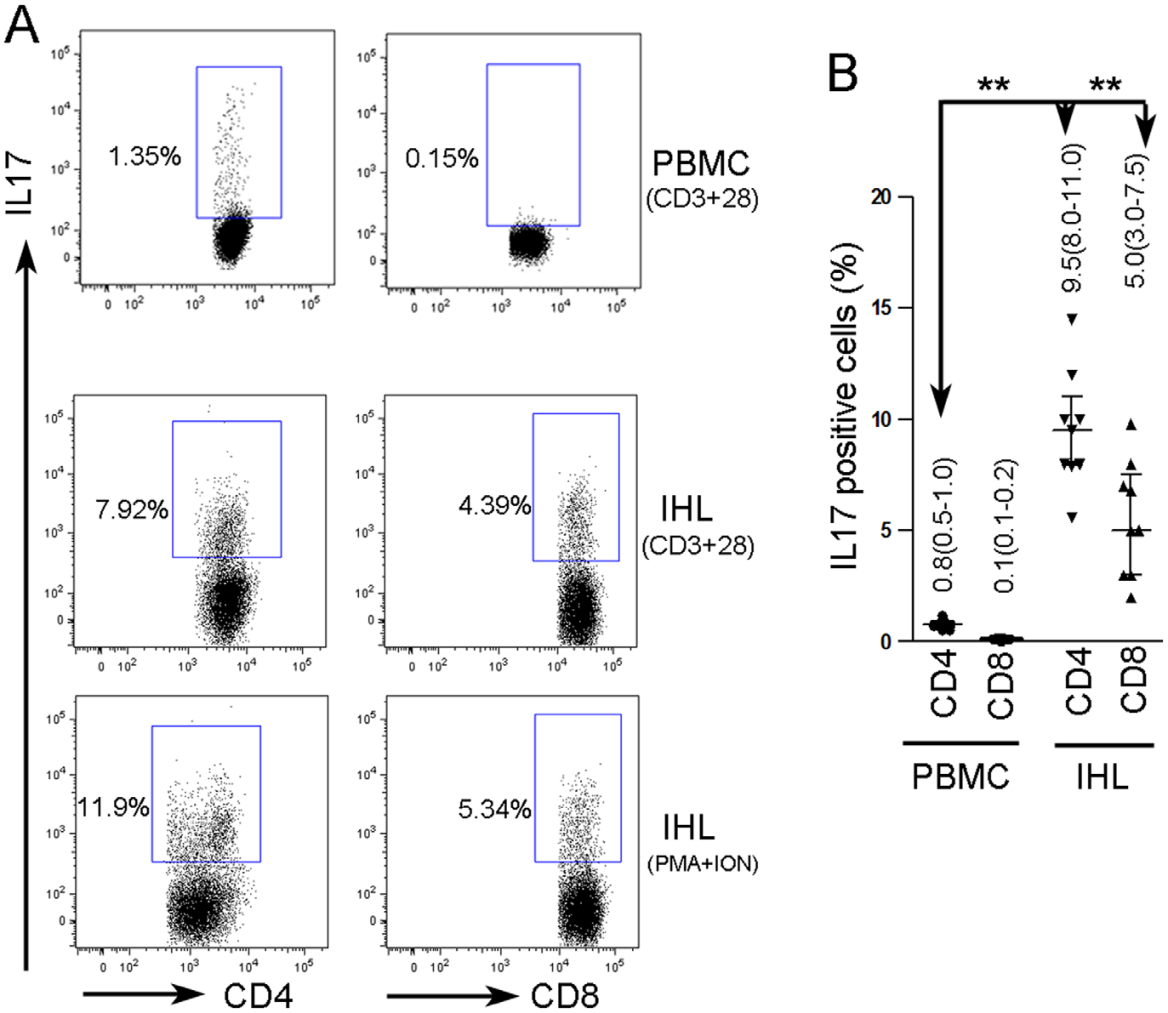
Intracellular IL17 staining in PBMCs and IHL of HCC patients. A: IL17 staining in PBMCs or IHL isolated from one representative of 9 independent HCC patients stimulated with anti-human-CD3 and CD28 (CD3+CD28). As parallel, IHL in 3 of the HCC patients were stimulated with PMA and Ionomycin (PMA+ION) were used to confirm the presence of IL17-producing T cells. Analysis was based on CD3^+^ gating (gating strategy is provided in Figure S2). B: Percentage of IL-17^+^ cells in the indicated cell populations. Each dot represents one of HCC patient. PBMCs: peripheral blood monoculear cells; IHL: intrahepatic lymphocytes. PMA: phorbol 12-myristate 13-acetate, ION: Ionomycin. **indicates *P<*0.001.

In order to identify the IL17-producing cells in the condition of HCC, which is reported to be one of the Th17 representative cytokines [13], [26], we obtained tumor-adjacent liver tissues and matched peripheral blood from 9 independent HCC patients. Using intracellular cytokine staining and FACS analysis we failed to identify any tumorous or hepatic cells (CD45-negative cells) that produce IL17 (data not shown). In the portion of intrahepatic lymphocytes (IHL), 9.5% (IQR: 8.0–11.0%) of CD3^+^CD4^+^T cells, and5.0% (IQR: 3.0–7.5%) of CD3^+^CD8^+^ T cells, were IL17-positive. (The gating strategy is provided in Figure S2). In the matched PBMCs, there were 0.8% (IQR: 0.5–1.0%) of CD3^+^CD4^+^ T cells identified as IL17-positive cells (Figure 2A, 2B). Infiltrated intrahepatic T cells were found to be the mainly IL17-producing cells.

### Proliferation of Liver Cancer Cells was Augmented in Presence of IL17-producing T-cells

**Figure 3:**
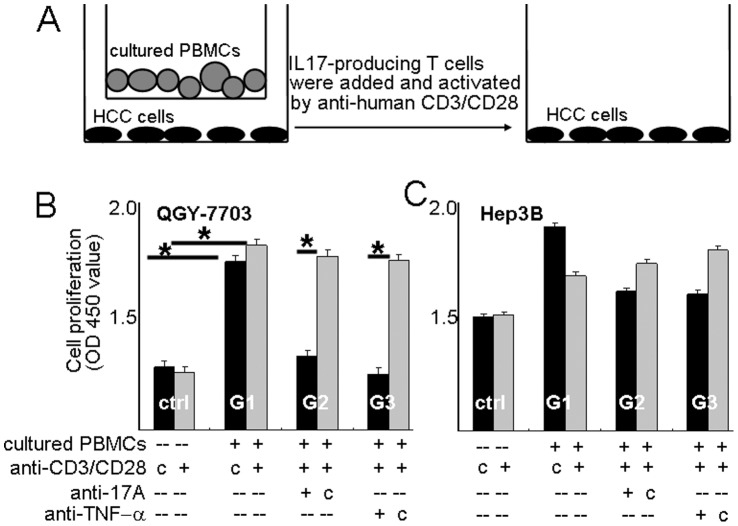
Proliferation of hepatocellular carcinoma cells in presence of activated IL17-producing T cells. A: Diagram of the co-culture experiments. B, C: The proliferation of HCC cells, QGY-7703 or Hep3B cell lines, was determined using a CCK8 reagents after being co-cultured with IL17-producing T cells for 48 h. PBMCs were isolated from the HCC patients and cultured with recombinant human IL-23 in the presence of plate-bound anti-human CD3 and anti-human CD28 for 7–10 days. IL17 production was confirmed by intracellular staining (Figure S3). The proliferation of HCC cells (ctrl) containing anti-human CD3 and anti-CD28 antibodies in the medium but without adding cultured T cells in the upper chambers was used as control. All the antibodies used here were 5 µg/ml. Letter “c” represents isotype control. The upper chambers contained T cells were removed before CCK8 reagents were added. Triplicates were performed for each of the treatment. *: *P<*0.05. Data shown is representative one from 3 independent experiments.

As the IL17-producing T-cells infiltrated in the tumor adjacent liver tissues, we assumed that these T-cells in the remnant liver tissue might affect the behavior of trace amount of tumor cells after hepatectomy. We studied HCC cell proliferation (as depicted in Figure 3A) by using two HCC cell lines, QGY-7703 and Hep3B cells in presence of IL17-producing T-cells, which were generated *in vitro* from HCC patient PBMCs (data were presented in Figure S3). Compared with QGY-7703 HCC cells only (Figure 3B, ctrl) cell proliferation increased significantly when they were co-cultured with the IL17-producing T-cells (Figure 3B, G1) (*P<*0.05). After neutralizing antibody against human IL17A (Figure 3B, G2) or TNF-α (Figure 3B, G3) was included in the culture system, the proliferation capacity of this HCC cells decreased significantly (*P<*0.05), the OD values showed similar to that of QGY-7703 cells alone. Effect of IL17 or TNF-α generated from activated T-cells in augmenting QGY-7703 proliferation was blocked (Figure 3B).

The proliferation of another HCC cell line, Hep3B (Figure 3C, G1) also showed increase after being co-cultured with the IL17-producing T-cells but with no statistically significant compared with the cells along (Figure 3C,ctrl). When anti-IL17A (G2) or anti-TNF-α (G3) neutralizing antibody was included in the medium, cell proliferation was weakly affected.

## Discussion

HBV-related HCC generally develops in a background of liver fibrosis or cirrhosis with extensive lymphocyte infiltration in the tissues. As is consistent with the literature [1], [5], our study showed that tumor size of greater than 5 cm in diameter significantly increased the risk of HCC early recurrence after curative resection. In addition, we found that an elevated level of serum IL17 before surgery was significantly associated with high risk of HCC early recurrence after adjustment for clinicopathologic factors which were generally accepted as indicators of high risk of HCC recurrence. Elevated pretherapy serum IL17 was an independent risk factor for HCC early recurrence. Patients with bigger tumors and elevated serum levels of IL17 had the highest risk of early recurrence and poor OS as compared to those with only one of these or without any these factors. Pre-treament levels of serum IL17 might serve as an additional potential indicator for predicting super high-risk HCC patients.

Remarkable advances in surgical and imaging modalities over the last few decades have improved the progression of HCC patients [1], [2], [3]. These advances have elevated the 5-year survival rate to 70% if the patients were carefully selected [2]. With the help of improved imaging facilities it is now possible to completely remove the tumorous tissues during surgery from patients with well-preserved liver function (Child-Pugh class A) and single nodular asymptomatic tumors without vascular invasion [2]. With the advances in gene profiling technology, molecular analysis has found that specific gene signatures, expressed by the adjacent nontumorous liver, rather than the tumorous tissues, reasonably predicted the DFS and OS of HCC patients [8], [9]. How much noncancerous tissue should be removed depends on the location of the cancer. Generally, 1–2 cm away from the cancerous margin was recommended [19]. However, in our study no significant difference of HCC early recurrence was shown between patients with the surgical margin of either more or less than 2 cm.

In this study, we found that an increase serum IL17 level was not significantly influenced by the tumor intrinsic characteristics. IL17 is one of the Th17 representative secreted cytokines. In addition to IL17A, the Th17 cells produce many other cytokines and chemokines, including IL21, IL22, IL26, IL6, TNF, CCL20 etc [13]. Staining of intracellular cytokines demonstrated that CD3^+^CD4^+^ and CD3^+^CD8^+^ T cells in infiltrated IHL of the tumor adjacent liver tissues were the mainly IL17-producing cells. The proliferation of HCC cells, QGY-7703, increased significantly when they were co-cultured with activated IL17-producing T cells. This capacity diminished when T-cell secreted IL17A or TNF-α were being neutralized by functional antibodies. The proliferation of another HCC cell line, Hep3B, also showed increase after being co-cultured with the IL17-producing T-cells but with no statistically significant. Therefore, the tumor cells themselves, and some cytokines, including IL17, TNF-α generated from liver infiltrated T-cells in the tumor environment all contributed to early HCC recurrence.

It has been reported that IL17-producing CD4^+^ T cells in patients with chronic HBV infection exacerbated liver damage under the condition of chronic HBV infection [27]. The contribution of IL17 and Th17-related immunity during carcinogenesis has been demonstrated recently [28], [29], [30]. The intratumoral numbers of IL17-producing cells were reported positively correlated with microvessel density in HCC [31]. IL17A promotes HCC metastasis via NF-κB induced matrix metalloproteinases 2 and 9 expression [32] and Th17 type immune responses promote the progression of non-small-lung-cancer [33]. Therefore, in the local liver environment, presence of the IL17-producing T cells in the remnant liver tissues after curative resection might exacerbate liver damage [27] and/or favor HCC development by persistently generating tumor supporting or promoting cytokines as well. IL17 and IL17-producing T-cells provide potential molecular targets for preventing/blocking HCC early recurrence. Results from phase I and phase II clinical trials using neutralizing antibodies against TNF-α in patients with ovarian cancer have demonstrated the positive therapeutic effects by blocking the Th17 action [34].

## Supporting Information

Figure S1 **ROC curves of serum IL10 and IL17 to predict HCC early recurrence.** The area under the ROC curve (AUC) for IL17 is 0.824 (*P<*0.001), it is 0.662 (*P = *0.003) for IL10.
(TIF)

Figure S2 **The gating strategy for analysis of the IL17-producing cells in intrahepatic liver lymphocytes (IHL).** Human CD45-positive cells were further separated by anti-human CD3 staining. IL17-producing cells were analyzed based on the cell populations that were CD3^+^CD4^+^ or CD3^+^CD8^+^cells.
(TIF)

Figure S3 **IL17-production T cells generated from PBMCs of an HCC patients.** PBMCs were isolated from 10-ml of peripheral blood and cultured with 20 ng/ml of recombinant human IL-23 in the presence of 10 µg/ml of plate-bound anti-human CD3 and 1 µg/ml of anti-human CD28 for 7–10 days. The cells were analyzed for intracelluar staining of IL17A and IL10 on D7 from the cultures.
(TIF)

Table S1 **Associations between clinicopathological factors and the risk of early recurrence of HCC in the first cohort of 105 HCC patients.**
(DOC)
