# Supplementary figures and images for: Elevated Pretherapy Serum IL17 in Primary Hepatocellular Carcinoma Patients Correlate to Increased Risk of Early Recurrence after Curative Hepatectomy
Source: PLoS One. 2012 Dec 5;7(12):e50035. doi: 10.1371/journal.pone.0050035 (PMC3515597; doi:10.1371/journal.pone.0050035)

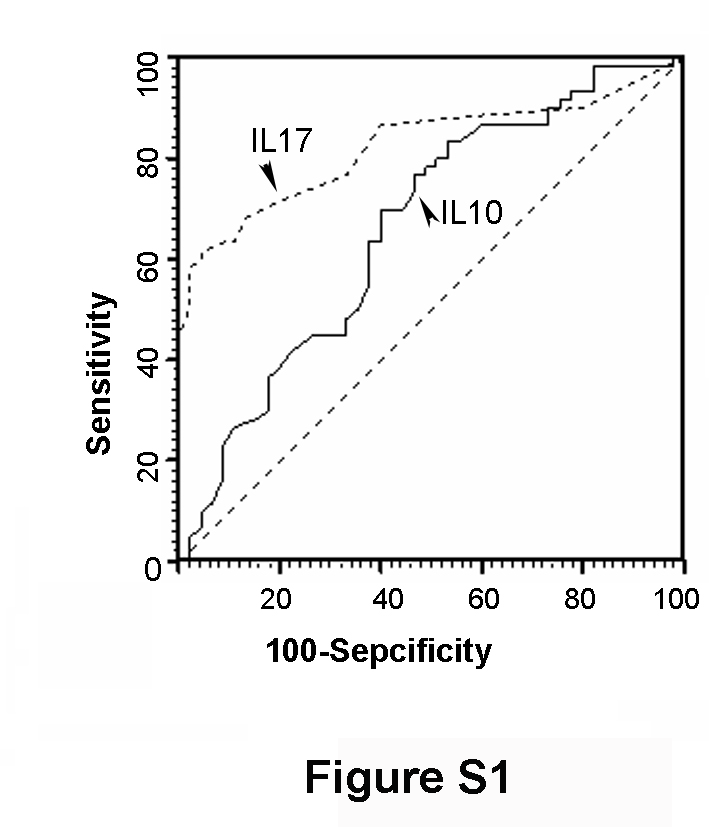

Supplement: Figure S1 — ROC curves of serum IL10 and IL17 to predict HCC early recurrence. The area under the ROC curve (AUC) for IL17 is 0.824 (P<0.001), it is 0.662 (P = 0.003) for IL10. (TIF) [file pone.0050035.s001.tif]

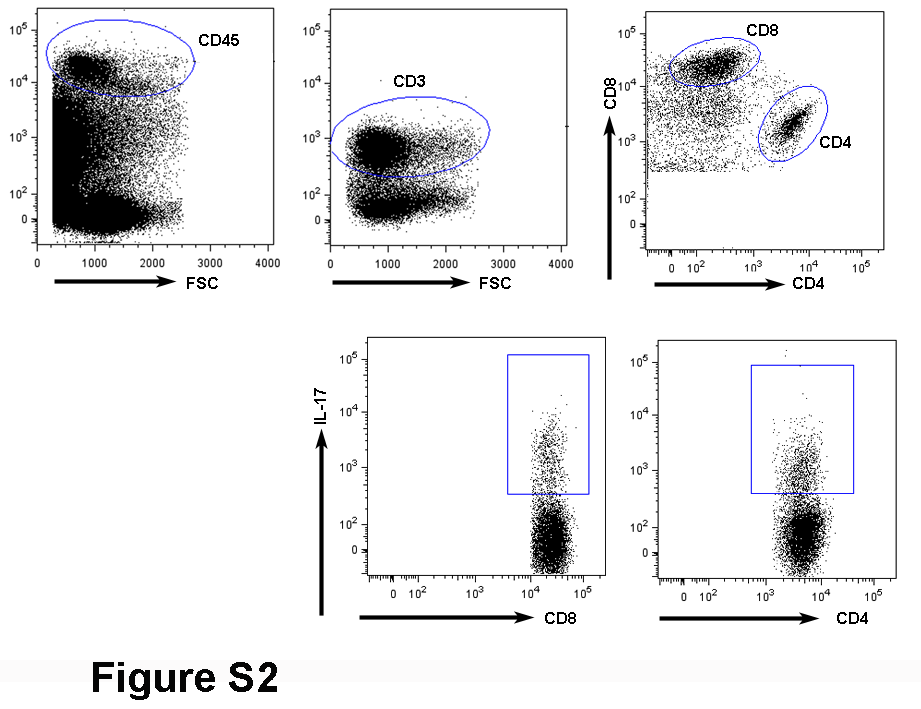

Supplement: Figure S2 — The gating strategy for analysis of the IL17-producing cells in intrahepatic liver lymphocytes (IHL). Human CD45-positive cells were further separated by anti-human CD3 staining. IL17-producing cells were analyzed based on the cell populations that were CD3+CD4+ or CD3+CD8+cells. (TIF) [file pone.0050035.s002.tif]

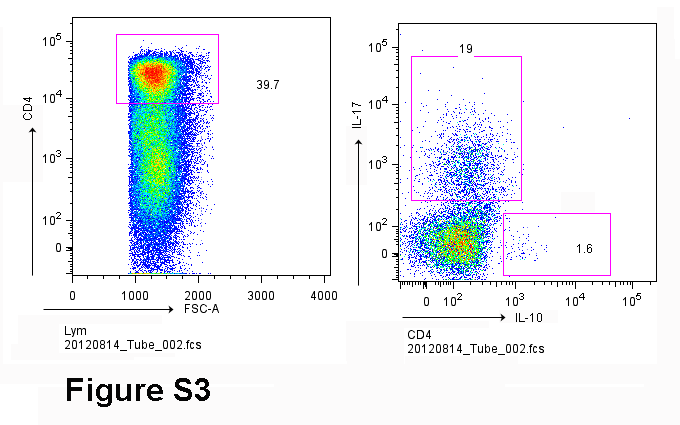

Supplement: Figure S3 — IL17-production T cells generated from PBMCs of an HCC patients. PBMCs were isolated from 10-ml of peripheral blood and cultured with 20 ng/ml of recombinant human IL-23 in the presence of 10 µg/ml of plate-bound anti-human CD3 and 1 µg/ml of anti-human CD28 for 7–10 days. The cells were analyzed for intracelluar staining of IL17A and IL10 on D7 from the cultures. (TIF) [file pone.0050035.s003.tif]
