# Supplementary material for: Elevated Pretherapy Serum IL17 in Primary Hepatocellular Carcinoma Patients Correlate to Increased Risk of Early Recurrence after Curative Hepatectomy
Source: PLoS One. 2012 Dec 5;7(12):e50035. doi: 10.1371/journal.pone.0050035 (PMC3515597; doi:10.1371/journal.pone.0050035)
Supplement: Table S1 — Associations between clinicopathological factors and the risk of early recurrence of HCC in the first cohort of 105 HCC patients. (DOC) [file pone.0050035.s004.doc]

Table S1. Associations between clinicopathological factors and risk of HCC early recurrence

| Category | Subcategory | Recurrence | Non-recurrence | Crude HR ^a^ |
| --- | --- | --- | --- | --- |
| Age (year) | median (IQR) | 52 (45-58) | 55 (48-64) | 0.99 (0.96-1.01) |
| Gender | Female | 8 | 6 | 1.00 |
|  | Male | 52 | 39 | 0.90 (0.43-1.91) |
| Serum HBV-DNA | <10000 copies/mL | 32 | 24 | 1.00 |
|  | ≥10000 copies/mL | 28 | 21 | 0.90 (0.54-1.51) |
| ALT | <40 U/L | 36 | 31 | 1.00 |
|  | ≥40 U/L | 24 | 14 | 1.48 (0.65-3.34) |
| Total bilirubin | <17.1 uM/L | 46 | 38 | 1.00 |
|  | ≥17.1 uM/L | 14 | 7 | 1.65 (0.61-4.51) |
| Albumin | ≥40 g/L | 36 | 31 | 1.00 |
|  | <40 g/L | 24 | 14 | 1.48 (0.65-3.34) |
| Surgical margin | ≥2 cm | 45 | 32 | 1.00 |
|  | <2 cm | 15 | 13 | 0.82 (0.34-1.96) |
| Resection | Minor | 44 | 36 | 1.00 |
|  | Major | 15 | 10 | 0.72 (0.29-1.78) |
| Blood transfusion | No | 41 | 36 | 1.00 |
|  | Yes | 19 | 9 | 1.44 (0.83-2.49) |
| BCLC classification | 0/A | 48 | 39 | 1.00 |
|  | B | 12 | 6 | 1.28 (0.68-2.40) |
